# Cotton functionalized with polyethylene glycol and graphene oxide for dual thermoregulating and UV-protection applications

**DOI:** 10.1038/s41598-023-31415-z

**Published:** 2023-04-11

**Authors:** Amit Kumar, Imen Kebaili, Imed Boukhris, Rahul Vaish, Anuruddh Kumar, Hyeong Kwang Benno Park, Yun Hwan Joo, Tae Hyun Sung

**Affiliations:** 1 School of Engineering, Indian Institute of Technology Mandi, Mandi, Himachal Pradesh 175005 India; 2 Department of Physics, Faculty of Science, King Khalid University, P.O. Box 9004, Abha, Saudi Arabia; 3 Groupe de Physique des Matériaux LuminescentsLaboratoire de Physique AppliquéeFaculté des Sciences de Sfax, Département de Physique, Université de Sfax, BP 1171, 3018 Sfax, Tunisia; 4 Laboratoire des Matériaux Composites Céramiques et Polymères (LaMaCoP), Département de Physique, Faculté des Sciences de Sfax BP 805, Université de Sfax, 3000 Sfax, Tunisia; 5 Center for Creative Convergence Education, Hanyang University, Seoul, 04763 South Korea; 6 Department of Electrical Engineering, Hanyang University, 222, Wangsimni-ro, Seongdong-gu, Seoul, 04763 Korea

**Keywords:** Energy storage, Polymer chemistry, Surface chemistry

## Abstract

A thermoregulating smart textile based on phase change material (PCM) polyethylene glycol (PEG) was prepared by chemically grafting carboxyl-terminated PEG onto cotton. Further deposits of graphene oxide (GO) nanosheets were made on the PEG grafted cotton (PEG-g-Cotton) to improve the thermal conductivity of the fabric and to block harmful UV radiation. The GO-PEG-g-Cotton was characterized by Attenuated total reflectance-Fourier transform infrared spectroscopy (ATR-FTIR), Raman spectroscopy, X-ray diffraction (XRD), x-ray photoelectron spectroscopy (XPS), and field emission-scanning electron microscopy (FE-SEM). With an enthalpy of 37 and 36 J/g, respectively, the DSC data revealed that the functionalized cotton's melting and crystallization maxima occurred at 58 °C and 40 °C, respectively. The thermogravimetric analysis (TGA) presented that GO-PEG-g-Cotton was thermally more stable in comparison to pure cotton. The thermal conductivity of PEG-g-Cotton increased to 0.52 W/m K after GO deposition, while pure cotton conductivity was measured as 0.045 W/m K. The improvement in the UV protection factor (UPF) of GO-PEG-g-Cotton was observed indicating excellent UV blocking. This temperature-regulating smart cotton offers a high thermal energy storage capability, better thermal conductivity, thermal stability, and excellent UV protection.

## Introduction

Smart textiles react in response to external stimuli or variations in environmental conditions. These are prepared by fusing smart elements like thermo-electric, ferroelectric, thermo-regulated, or shape memory alloys with the textile substrate^1^. The incorporation of these smart elements in textiles can help in drug delivery, energy harvesting, body protection, artificial intelligence, energy storage, antibacterial, and other applications. In recent years, the demand for thermoregulating and UV-protective clothing has increased due to changes in solar heat balance and the depletion of the ozone layer. The thermoregulated textiles are widely used in different applications like space, such as suits and gloves; sportswear, such as snowboard gloves, underwear for cycling, ski boots, footwear, and golf shoes; the medical field, such as bandages; protective textiles, such as helmets and firefighter's suits; containers and packaging; automotive textiles; building materials; and so on^2–6^.

Phase change material (PCM) incorporated textiles are considered the most viable option for the preparation of thermoregulating smart textiles. PCM serves as a thermal buffering agent and responds immediately to temperature changes in both the environment and human body parts^7^. When PCM-incorporated textile is exposed to a hot environment, the PCM absorbs and stores heat by melting. This ability of PCM to absorb heat keeps the textile material at a constant temperature. The accumulated energy is released into the environment during solidification as the ambient temperature drops^8^. PCMs can be categorized into three groups based on the phase change state: solid–liquid, solid–solid, and liquid–gas PCMs. The solid–liquid PCMs suffer from the leakage problem, while liquid–gas PCMs do not practically exist due to high volumetric change. There is a need for a technique or a process that directly grafts PCM molecules onto polymer chains. The product must be biodegradable, ecologically stable, and safe for use in repeated cycles without developing shape deformities. In solid–solid PCM (S-SPCM), no liquid or gas generation takes place. This eliminates the need for encapsulation or extra reservoirs, which were necessary for the other two categories of PCM, and thus, lowers the cost of the system. The other characteristic features of S-SPCM include non-corrosiveness, better design flexibility, good thermal stability, non-toxicity, and durability^9,10^. S-SPCMs thermoregulating cloth can be made using either a physical approach or a chemical approach. Through coating, laminating, mixing, soaking, adsorbing, or spinning, PCM is applied to textiles using the physical approach^11^. The physical approach has cyclic stability issues. Phase segregation happens after several repeated cycles, which also results in a reduction in the PCM's capacity to retain heat. PCM is linked to some polymer components of textile material either by cross-linking, block formation, or grafting in the chemical approach. The polymer component acts as a solid framework and remains in a solid state during the phase change, while PCM stores or releases energy^12^. Polyethylene glycol (PEG) is one of the available PCMs that is often employed as a heat storage medium for the preparation of S-SPCMs. PEG has unique advantages such as high latent heat of fusion, simple reagent reactivity, cyclic stability, non-toxicity, and availability in a range of molecular weights with varying melting and cooling enthalpies^13–17^.

The incorporation of PCM in textiles has been documented by numerous researchers. Shin et al. (2005) prepared thermo-regulating eicosane microcapsules-based polyester knit fabric by pad-dry-cure technique. The fabric was capable to absorb 4.44 J/g of heat^18^. Wet spinning was employed by Zhang et al. (2006) to create thermo-regulated poly acrylonitrile vinylidene chloride fibers. The n-octadecane and paraffin microcapsules were prepared with urea–melamine–formaldehyde copolymer followed by wet-spinning. The fibers with a 40% weight percentage of PCM displayed a melting enthalpy of 44 J/g. Gao et al. (2009) reported melt spun acrylonitrile–methyl acrylate copolymer containing *n*-octadecane microcapsules. Nejman and Cieślak (2017) coated polyester knitted and woven fabrics with n-hexadecane and n-octadecane paste. The phase change enthalpy reported in the knitted and woven fabrics was 56–60 and 25–28 J/g, respectively. Lu et al. (2019) employed electrospinning for developing a smart cloth using paraffin wax and polyacrylonitrile. The cesium tungsten bronze was used for near-IR absorption and the modified fabric possessed latent heat of 60.31 J/g^22^. Wang et al. (2021) also used electrospinning to prepare PEG and titanium dioxide (TiO_2_) based smart textiles^23^. PEG was used as PCM while TiO_2_ served the purpose of UV absorption. All of these published findings demonstrated the use of a physical strategy as far as the textile substrate is concerned. There is a need to chemically graft PCM on the textile surface such that it is linked to the substrate either by cross-linking, block formation, or grafting. With the change in the surrounding temperature, the polymer/textile component will act as a solid framework during the phase change and remains in a solid form, whereas PCM can easily store or release energy.

Cotton is the industry leader in textiles due to its distinctive qualities, which include good moisture absorption, comfort in wear and handling, maximum strength, resistance to alkalis, and good shear characteristics like a drape. The repeating unit of cotton is cellobiose, which has an empirical formula (C_12_H_20_O_10_)_n_ and its degree of polymerization (n) ranges from 2500 to 5000^24^. Cotton is best suited for chemical grafting because each anhydroglucose unit of cellobiose has the available reactive sites or hydroxyl groups.

Cotton and PEG side chains are both organic materials with limited thermal conductivity, hence their ability to absorb heat from a hot environment is fairly subpar. A functionalized form of graphene, known as graphene oxide (GO), has several polar groups that are easily attracted to the surfaces of fibers. GO has unique properties such as stimulus reactivity, UV absorption, hydrophobicity, high electrical conductivity, non-toxicity, and non-corrosiveness, and has attracted interest in textile goods for a variety of uses, including electronic textiles, medical textiles, and advanced composites^25,26^. Cai and coworkers coated cotton fabric with GO using the dip-dry method^27^. The coated fabrics demonstrated excellent electrical conductivity, surface hydrophobicity, and UV protection qualities. Shathi et al. (2020) coated GO on textile by pad-cure to produce highly pliable and launderable sports bra for tracking human health while Shateri-Khalilabad and Yazdanshenas (2013) deposited GO on cotton through dipping to prepare electro-conductive fabric^28,29^. The deposition of GO on cotton improves the fabric's thermal conductivity, resulting in higher heat uptake from the environment and shielding the body by blocking harmful UV light. Zhang et al., (2021) reported the incorporation of n-eicosane PCM into multilayer graphene through a vacuum implementation technique^30^. A composite of n-eicosane with Fe_3_O_4_ and silica shell was prepared by Do et al., (2021) and later on Cu nanoparticles were loaded on the composite to increase its thermal conductivity^31^. The prepared composite demonstrated better latent heat enthalpy and crystallization ratio, and high thermal stability.

In the present work, PEG was chemically grafted onto cotton by taking the advantage of hydroxyl groups present in the cellulose. The prepared smart cotton samples were referred to as PEG-g-Cotton. The graphene oxide (GO) nanosheets were deposited on the surface of PEG-g-Cotton to improve its thermal properties and enhance its utility as ultra-violet protective textile. The GO-PEG-g-Cotton was examined by Attenuated total reflectance-Fourier transform infrared spectroscopy (ATR-FTIR), Raman spectroscopy, X-ray diffraction (XRD), X-ray photoelectron spectroscopy (XPS), and field emission-scanning electron microscopy (FE-SEM). To evaluate the fabric's heating and cooling enthalpies as well as its thermal durability, DSC and TGA measurements were made. The other textile methodologies were used for measuring modified cotton fabric mechanical properties, thermal conductivity, ultra-violet protection factor (UPF), and end-use durability.

## Materials and methods

### Materials

PEG (6000 average molecular weight), maleic anhydride (MA), titanium isopropoxide (catalyst), cerric ammonium nitrate (CAN), triphenylphosphine (PPh_3_), and glycidyl methacrylate (GMA) were purchased from Sigma-Aldrich. Toluene, hydrochloric acid (HCl), and sodium hydroxide (NaOH) were obtained from Fisher Scientific. GO was purchased from Shilpent, India and pure cotton in fiber as well as fabric form (40*40 Ne), was provided by Vardhman Industries, Himachal Pradesh, India.

### Method

Pure cotton, both in fiber as well as fabric form, was treated with 18% NaOH at 25 °C for 1–2 min before being washed with distilled water and then subjected to further treatment with 0.01 N HCl for neutralization of NaOH. Further, cotton was again washed thoroughly with distilled water, dried for an additional night in a hot air oven at 45 °C, and weighed^32^. Cellulosic fiber swells after being treated with NaOH, which improves its absorption capacity.

In the first step, 15.4 g (2 mol) MA was added to 5 g (1 mol) PEG along with 2–3 drops of titanium isopropoxide as the catalyst to form carboxyl terminated PEG in a round bottom flask coupled with the dean and stark apparatus. The reaction mixture was stirred and heated at 180 °C by using a magnetic stirrer under a nitrogen atmosphere for 12 h^33^. In the second step, 1 g of dried cotton fiber was treated with 5 g of GMA (taken in excess) along with a very small amount of CAN (1 mg) in 100 ml of Toluene for 12 h under a nitrogen atmosphere at 100 °C to form cellulose/glycidyl methacrylate copolymer.

In the third step, carboxyl terminated PEG (taken in excess) was grafted on 1 g cellulose/glycidyl methacrylate copolymer by using PPh_3_ (1%) as a catalyst in 100 ml of Toluene for 12 h at 100 °C in a nitrogen environment.

**Scheme 1:**
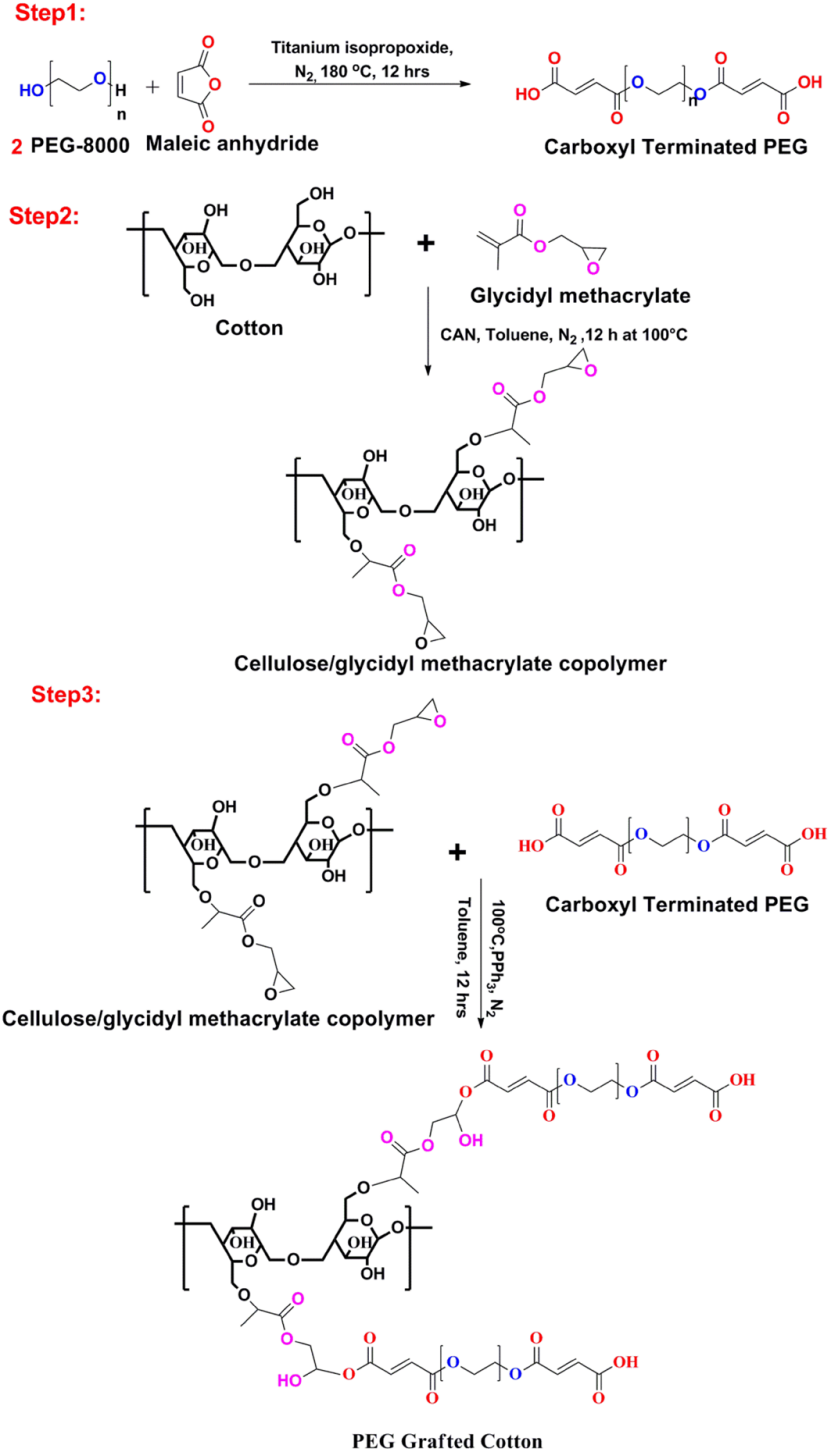
Schematic representation of the synthesis of PEG-g-Cotton.

After completion of the chemical reaction as presented in Scheme 1, PEG grafted cotton fiber (PEG-g-Cotton) was dried and the final weight was calculated. The grafting percentage was determined by using the formula^34^:

$${\text{Grafting Percentage}} = \, ({\text{Final weight of PEG - g - Cotton}} - {\text{Initial weight of NaOH treated cotton}}) \, \times \, 100/\left( {\text{Initial weight of NaOH treated cotton}} \right).$$

The same process was repeated for the preparation of PEG-g-Cotton fabric samples and the grafting percentage was calculated.

The GO solution was prepared by dissolving 1 g GO in 1L of deionized water and then employing an ultrasonic probe for 1 h. Thereafter, the unexfoliated GO particles were removed by centrifuge technique. PEG-g-Cotton was submerged in the prepared GO solution for 2 h for loading the GO particles, followed by sonication for 24 h. Once the unexfoliated GO was removed from the modified cotton (GO-PEG-g-Cotton), it was washed with deionized water and dried for 24 h at 45 °C in oven^35^.

### Characterization

FTIR measurements were performed by Spectrometer-2 (Perkin Elmer) instrument using the ATR technique with 50 scans per sample. A Raman spectrometer (Horiba Lab RAM HR evolution) was used to obtain Raman spectra. XRD experiments were carried out on Powder X-ray Diffractometer (Rigaku Corporation Smart Lab). A high-resolution transmission electron microscope (HR-TEM; FP 5022/22-Tecnai G2 20 S-TWIN) was employed for GO particle analysis. Thermofisher scientific X-ray photoelectron spectrometer was used for the cotton samples XPS analysis. The morphology of cotton samples and GO was investigated by using FE-SEM (JFEI SEM-450) with an energy-dispersive x-ray spectrometer. DSC and TGA analysis were done by using the STA-6000 (Perkin Elmer) instrument. The DSC study was performed at temperatures between 0 and 100 °C at 10 °C/min heating and cooling rates, respectively in a nitrogen atmosphere. The dried samples weighing about 10 mg were heated from 30 to 500 °C at a rate of 10 °C/min while flowing 20 ml/min of nitrogen for TGA analysis. The thermal conductivity of the samples was analyzed (Testex TF130) using a flat plate thermal conductivity meter. The UPF was calculated using a UV spectrophotometer (Perkin Elmer UV–vis Spectrophotometer). According to Australian/New Zealand Test Standard AS/NZS 4399:1996, the fabric's UPF rating of 15 to 24 suggests reasonable protection, 25 to 39 indicates very good protection, and 40 to 50 and more indicates outstanding protection^36,37^. The sample GSM (Weight in grams per square meter) was calculated using a GSM cutter by the American Society for Testing and Materials (ASTM) D-3775 standard. The Grab test technique was utilized to measure the fabric's tensile strength according to ASTM D-5034, and the fabric's abrasion resistance was evaluated in accordance with ASTM D-4685 using a Martindale abrasion resistance tester. The prepared samples were washed for 20 washing cycles by following ISO 105-C10:2006 standard.

## Result and discussion

### Characterization of PEG, GO, and GO-PEG-g-Cotton

**Figure 1:**
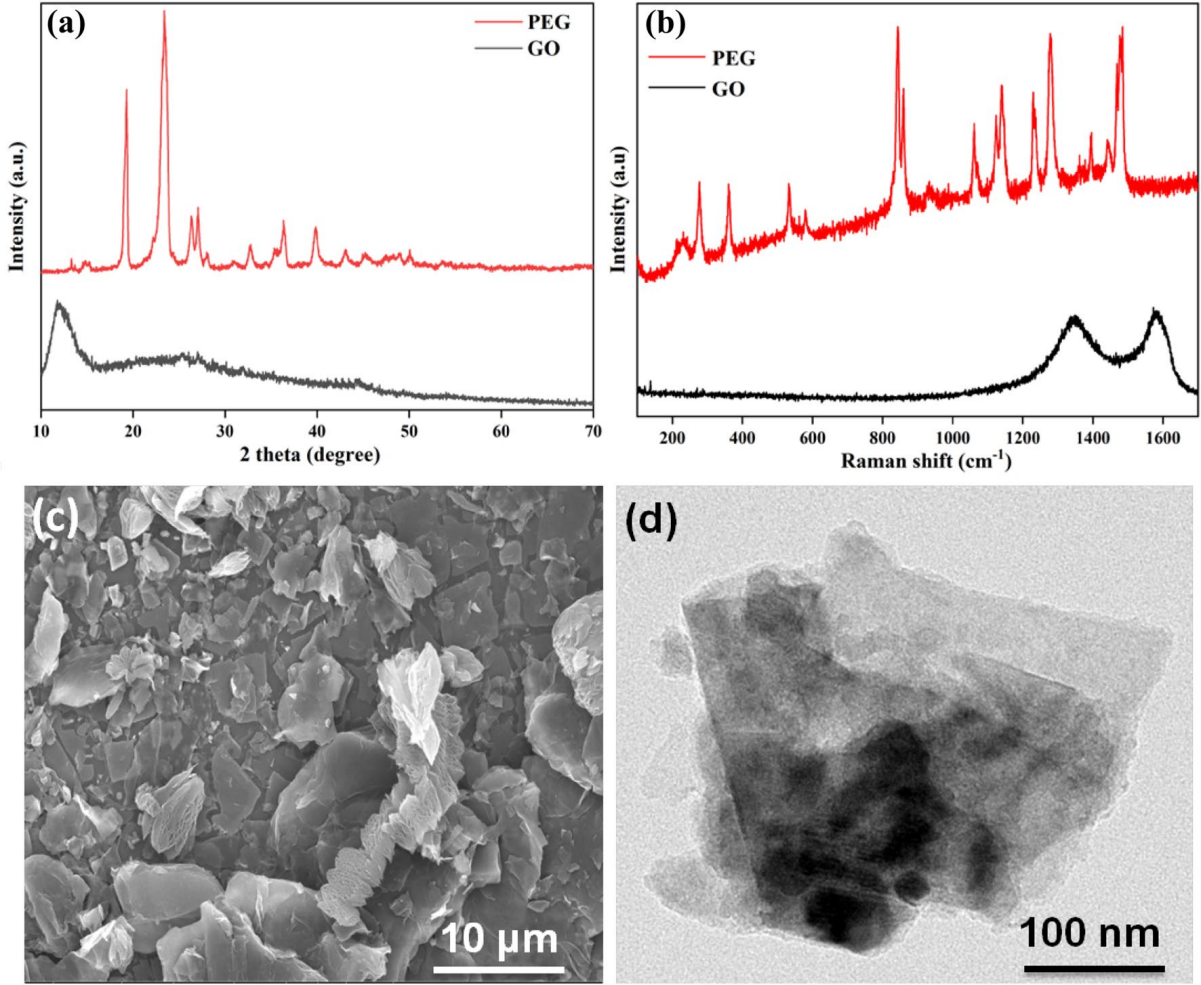
(**a**) XRD of PEG and GO, (**b**) Raman spectrum of PEG and GO, (**c**) FE-SEM of GO, and (**d**) TEM of GO.

Figure 1 presents the characterization of PEG and GO used for the cotton modification. The XRD peaks observed in PEG at 2θ = 13.3°, 14.71°, 15.18°, 19.31°, 23.37°, 26.33°, 26.95°, 27.96°, 30.89°, 32.73°, 36.26°, 39.88°, and 43.13° matched the standard JCPDS No. 49-2097 perfectly. The XRD of GO showed a wide diffraction peak at 2θ = 11.6°, corresponding to the (001) crystallographic plane^38^. The PEG Raman lines at 841 and 858 cm^−1^ presented –CH_2_ rocking and (C–O & C–C) stretching vibrations. The bands at 1123 and 1141 cm^−1^ were attributed to the –CH_2_ twisting vibrations of trans- and gauche- conformations relative to the C–O bond, respectively, while the bands at 1229 and 1279 cm^-1^ were attributed to the –CH_2_ wagging vibrations of trans- and gauche-conformers relative to the C–C bond, respectively. The Raman lines from 1440 to 1484 cm^−1^ belong to δ(O–C–C) and δ-CH_2_ scissoring characteristics. In the spectral range 1600–2600 cm^−1^, PEG Raman lines remain inactive while the spectral lines between 2800 and 3000 cm^−1^ are assigned the methylene group C–H stretching vibration^39,40^. The GO Raman spectra presented bands at 1586 cm^−1^ and 1348 cm^−1^, which represent the graphene G band and D band, respectively^28^. Figure 1c,d presents the FE-SEM and TEM images of GO, which illustrate its nanosheet forms.

**Figure 2:**
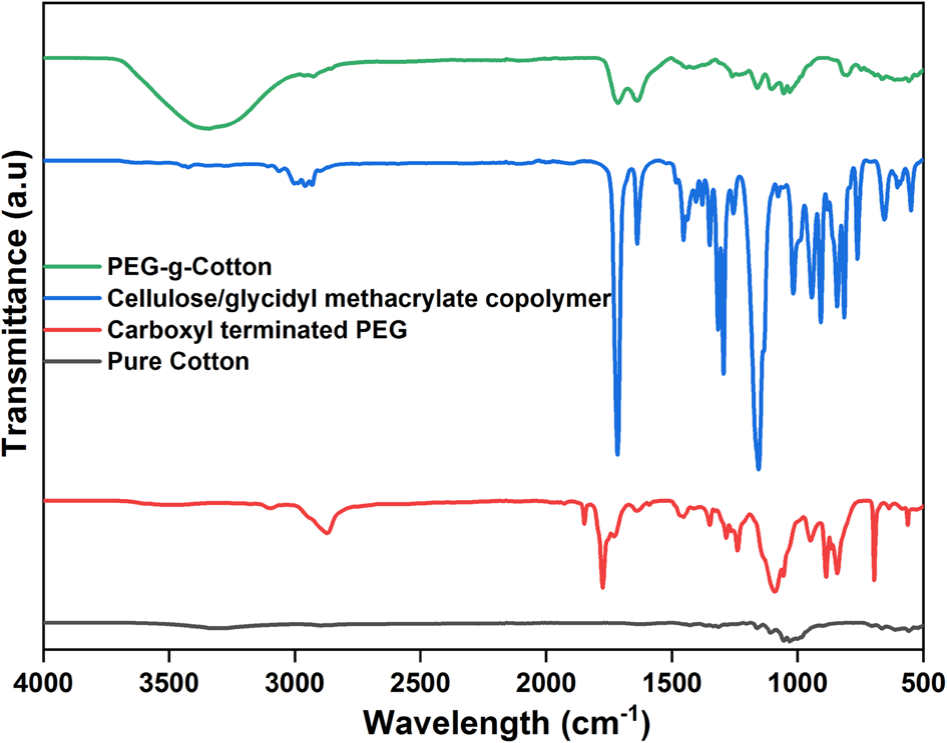
FTIR spectra of prepared samples.

The percentage of PEG grafted on cotton was obtained to be nearly 17% while 4% of the weight of GO was observed to be deposited on the PEG-g-Cotton. The FTIR spectra of pure cotton, carboxyl terminated PEG, cellulose/glycidyl methacrylate copolymers, and PEG-g-Cotton are shown in Fig. 2. ATR-FTIR spectrum of the cotton reveals a prominent band at 3333 cm^−1^ which corresponds to hydroxyl (OH) groups in cellulose and water. The stretching vibration of C-H in cellulose is correlated to the band at 2891 cm^−1^, while the presence of moisture in cotton is attributed to the band at 1625 cm^−1^. The cellulose CH_2_ symmetric bending is associated with the band at 1428 cm^−1^, while the C–H and C–O groups bending vibrations are correlated with the bands at 1360 and 1316 cm^−1^, respectively. The C–O–C stretching of β-glycosidic linkage is attributed to the band at 1160 cm^−1^, while an intense band at 1030 cm^−1^ is observed due to the C–O stretching of C-6 in cellulose^41–43^. The formation of carboxyl terminated PEG was confirmed by carbonyl stretching (C=O) vibration of ester bond at 1720 cm^−1^ and absorption band observed at 3421 cm^−1^ which relates to the free –OH group of PEG^33^. The ester group in the cellulose/glycidyl methacrylate copolymer was responsible for the band at 1716 cm^−1^, and the free hydroxyl group of cellulose contributed to the peak at 3425 cm^−1^. Additionally, a small band found at 3059 cm^−1^ was associated with the copolymer's oxirane ring. In the case of PEG-g-Cotton, the band due to –OH stretching was more intense showing the incorporation of new O–H groups onto cotton. Further, new intense bands at 1637 cm^−1^ and 1714 cm^−1^ were assigned to the ester group of methacrylate and maleic anhydride components added during the grafting process. It was also observed that the intensity of bands around 1160 cm^−1^ (due to C–O–C stretching) increased due to the formation of new C–O–C linkages. The band at 1030 cm^−1^ (caused by C–O stretching of C-6 in cellulose) was less intense in PEG-g-Cotton because the sites were occupied by the grafted component. The results of FTIR confirm the grafting of PEG onto cotton.

**Figure 3:**
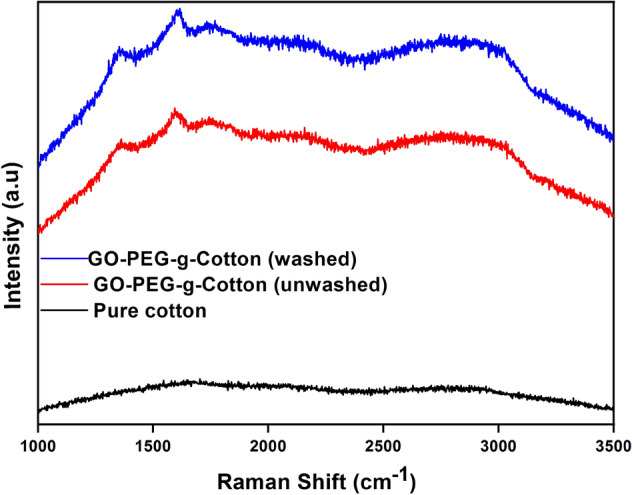
Raman spectra of pure cotton and GO-PEG-g-Cotton.

Figure 3 illustrates the Raman spectra of cotton samples. A peak observed in GO-PEG-g-Cotton between 2800 and 3000 cm^−1^ is assigned to the methylene group C–H stretching vibration of PEG^40^ but pure cotton exhibits no discernible peak^35^. This verifies the formation of PEG-g-Cot. Additionally, GO-PEG-g-Cotton displays two additional peaks at 1596 cm^−1^ and 1356 cm^−1^, which represent the graphene G band and D band, respectively^44^. This supports the deposition of GO on PEG-g-Cot. The Raman spectrum obtained after washing showed consistency with the unwashed one, indicating that PEG and GO remained present in the cotton polymer system even after 20 washing cycles.

**Figure 4:**
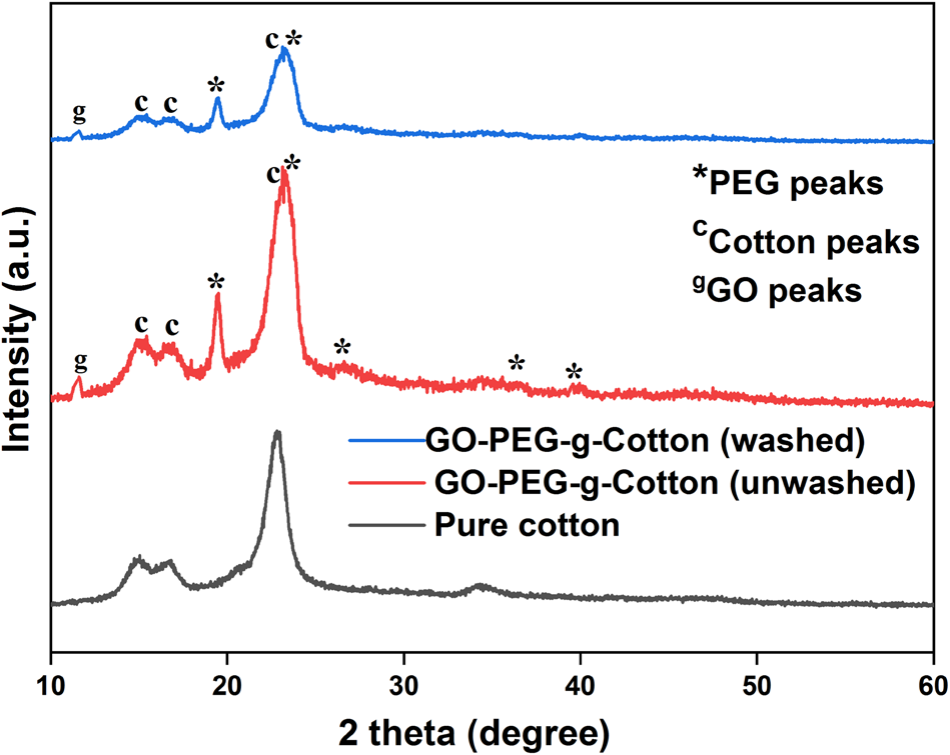
XRD plot of pure cotton and GO-PEG-g-Cotton.

Figure 4 shows the XRD plots of the pure cotton and GO-PEG-g-Cotton (before and after 20 laundry cycles). All the samples exhibited identical diffraction peaks at 2θ = 14.9°, 16.52°, 22.91°, and 34.58°, which are in agreement with Miller indices for cellulose Iβ and represent (110), (110), (200), and (004) crystallographic planes of cotton^35,45,46^. Since the PEG was chemically grafted on the cotton backbone, the peaks in GO-PEG-g-Cotton at 2θ = 19.43°, 23.31°, 26.40°, 36.288°, and 39.78° were attributed to PEG, which is consistence with the XRD peaks as presented in Fig. 1. A small peak corresponding to GO was observed at 2θ = 11.6°^44,47^. Additionally, the GO-PEG-g-Cotton XRD pattern after washing (20 cycles) was found consistent with the modified fabric that has not been washed. This signifies that a strong chemical bonding between PEG and cotton has been formed, and GO is still trapped inside the fiber polymer matrix with excellent washing endurance.

**Figure 5:**
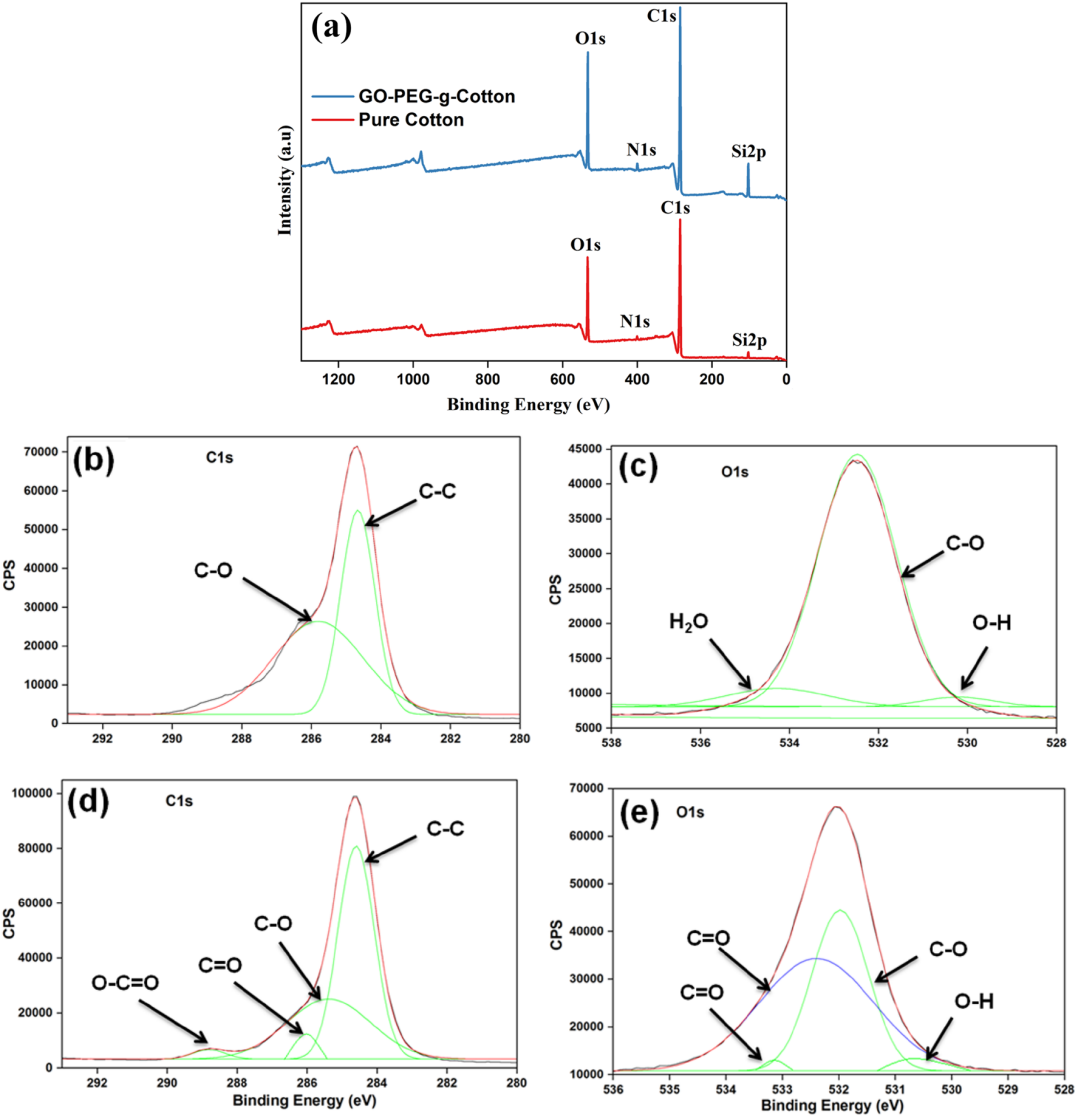
(**a**) XPS survey of pure cotton and GO-PEG-g-Cotton, (**b,c**) pure cotton high-resolution spectra of C 1s and O 1s, (**d,e**) GO-PEG-g-Cotton high-resolution spectra of C 1s and O 1s.

To further confirm the grafting of PEG and GO deposition on cotton XPS analysis for pure cotton and GO-PEG-g-Cotton was performed. Figure 5a shows the XPS survey spectrum of pure cotton and GO-PEG-g-Cotton. In both samples, carbon, oxygen, nitrogen, and silicon were observed as the main constituent elements^41,45,46^. During the processing of textiles (spinning/ weaving/chemical processing), different silicon-based softeners are applied to yarn or fabric to improve its smoothness and prevent it from being damaged by different machine parts. Although the pristine cotton sample used for the study was thoroughly washed, the remaining constituents of the silicon-based softener resulted in a Si peak. The usage of nitrogen-based fertilizers for cotton growing is responsible for the nitrogen's (N 1s) presence. Along with other proteins and cell debris, the nitrogen-based fertilizer molecule absorbs into the cotton fiber's lumen. All of these are absorbed on the cotton ball's surface or remain inside the cotton's lumen after it bursts. As presented in Fig. 5b, the C 1s peak of the pure cotton was deconvoluted using Gaussian fitting, and two peaks were found at 285.870 eV, and 284.65 eV, assigned to C–O and C–C bonds, respectively. Figure 5c presents the O 1s contributions at 534.39, 532.53 eV, and 530.31 eV which were attributed to H_2_O, C–O, and OH groups^48–51^. In GO-PEG-g-Cotton, the content of carbon, and oxygen got significantly increased as compared to pristine cotton. In the deconvoluted C 1s GO-PEG-g-Cotton spectrum as shown in Fig. 5d_,_ two new peaks associated with C=O and O–C=O bonds were seen, which were not observed in the same region of pure cotton. These peaks resulted due to the formation of ester linkages (PEG grafting) on the cotton surface and also due to GO deposition. In the O 1s spectrum of GO-PEG-g-Cotton as illustrated in Fig. 5e_,_ the formation of the C=O bond was confirmed at binding energy 532.43 eV.

**Figure 6:**
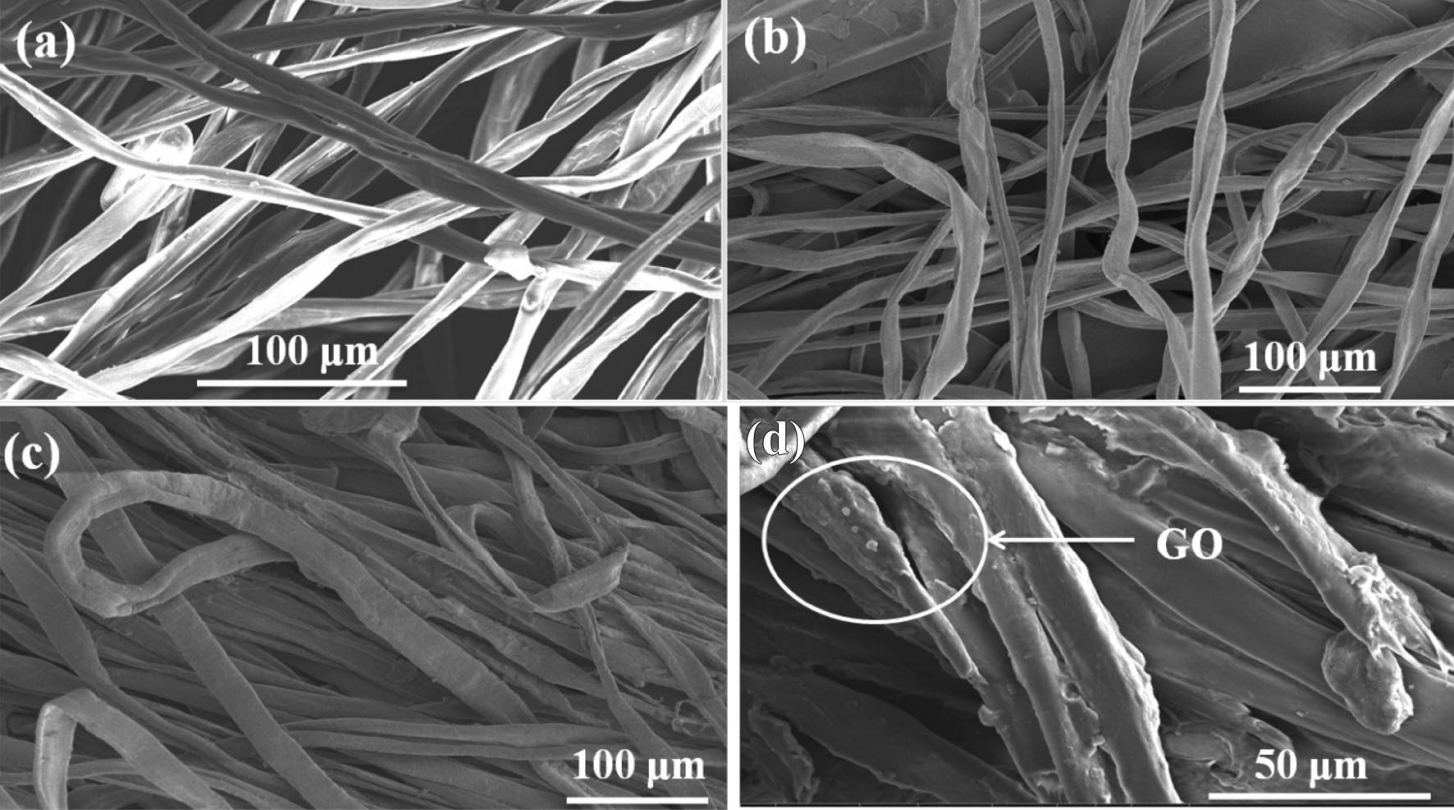
FE-SEM images (**a**) pure cotton, (**b**) NaOH treated cotton, (**c**) PEG-g-Cotton, (**d**) GO-PEG-g-Cotton (after washing).

Figure 6 presents the FE-SEM images of cotton samples for examining the surface morphologies. Pure cotton fibers were seen to be irregular, and uneven, and had a surface resembling a twisted ribbon as presented in Fig. 6a. As shown in Fig. 6b, NaOH causes cotton to swell, which improves its absorption ability. PEG-g-Cotton presented a smooth and cylindrical appearance as illustrated in Fig. 6c. GO nanosheets were loaded on the fabric surface by submerging PEG-g-Cotton in the prepared GO solution. As shown in Fig. 6d, GO nanosheets were observed to be uniformly deposited on the GO-PEG-g-Cotton even after washing. The grafting process effectively bonded the PEG on the cotton backbone and after 20 washing cycles, GO continued to be contained within the cotton polymer matrix.

Energy dispersive X-ray (EDX) (Supplementary Information) was also performed to depict the surface elemental composition of GO-PEG-g-Cotton. The carbon content of samples increases when GO was deposited on PEG-g-Cotton. The development of additional hydrogen bonds when cotton was washed results in a modest drop in the carbon content, as evidenced by the EDX mapping of modified cotton. Therefore, it is confirmed by the SEM and EDX data that GO particles were successfully deposited on the grafted cotton and remained adherent there even after 20 laundering cycles.

### Measurement of thermoregulating property of GO-PEG-g-Cotton

**Figure 7:**
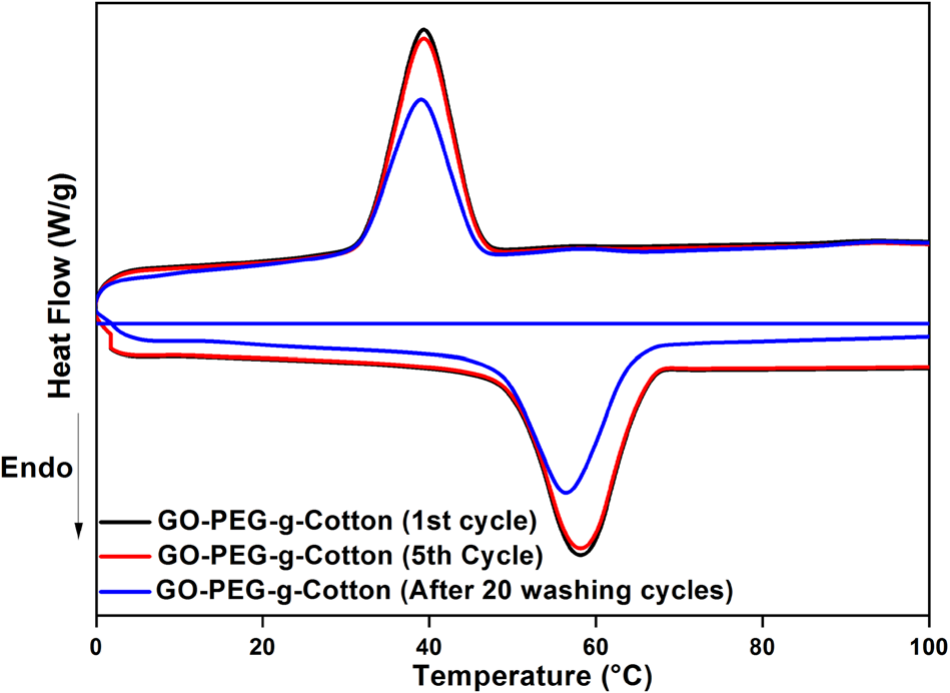
DSC thermogram of GO-PEG-g-Cotton.

The phase change characteristics of GO-PEG-g-Cotton were examined using the DSC thermogram illustrated in Fig. 7. Kumar et al. (2022) reported that PEG-6000 shows melting and cooling enthalpy of about 282 J/g and 142 J/g with the endothermic and recrystallization peak around 59 °C and 40°C^17,52^. In the present work, GO-PEG-g-Cotton phase change temperature (Tm) was found to be around 58 °C while the crystallization peak appeared at 40 °C for the first DSC heating and cooling cycle. Further, the melting and crystallizing enthalpies of GO-PEG-g-Cotton were calculated as 37 J/g and 36 J/g respectively. The measured enthalpies were lower than those of pure PEG because pure PEG only made up a small portion of the sample weight used for analysis. Due to the modified fiber's rapid cooling, crystallization is never complete, resulting in a significantly decreased crystallization enthalpy. The enthalpies measured during heating and cooling after the fifth repeated cycles were identical to those obtained during the first cycle. To ensure the cyclic stability of chemically grafted PCM, DSC analysis of GO-PEG-g-Cotton was carried out after 20 washing cycles. The melting and crystallizing enthalpies of washed cotton were found as 28 and 26 J/g, respectively, with melting around 56 °C while the re-crystallization temperature peak was observed at 39 °C. The fabric sample was largely unaffected by the washing procedure, allowing the grafting technology to be used for everyday clothing.

The thermoregulating property of the prepared fabric was attributed to the chemical grafting of PCM (PEG) on the cotton backbone. PEG was chemically attached to the cotton polymeric chain, which served as a solid framework. As the environment's temperature changes, PEG absorbs and releases heat, but does not come out due to covalent bonding, keeping the entire system in a solid state.

The outcomes of this investigation were compared with the available literature in Table 1. Researchers have attempted to enhance the comfort of textiles by incorporating PCM with physical approaches. PCM microencapsules were introduced on textile material by pad-dry-cure, coating, or spinning techniques. In these physical approaches, cyclic stability, re-crystallization, and lower enthalpies after washing remain an issue. As noted, the prepared cotton showed effective thermoregulating performance as compared to those reported in other studies in the literature.

**Table 1:** Literature for incorporation of PCM into textile.

Sr. No	Fibre used and PCM Application Method	PCM Used	Temperature (°C)		Enthalpy (J/g)		References
			Melting	Re-crystallization	Melting	Re-crystallization	
1	Polyester knit fabrics, pad-dry-cure	Eicosane	35	31.7	4.44	–	^18^
2	Polyacrylonitrile-vinylidene chloride, microencapsulation (wet spinning)	*n*-Octadecane and paraffin	29.8	21.5	44	30	^19^
3	Polyacrylonitril, microencapsulation (melt spinning)	*n*-Octadecane	27.3	20.2	25	21	^20^
4	Poly(lactic acid), pad-dry-cure	PEG	53	25	43	34	^53^
5	Polyester knitted and woven fabrics, coating	*n*-Hexadecane and *n*-octadecane	17	11	56–60 and 25–28	–	^21^
6	Polyacrylonitrile, coaxial electrospinning	Paraffin	38.32	35.23	59.26	59.05	^54^
7	Polyurethane, coating	Paraffin	46.5	35	236	235	^55^
8	Cotton	PEG	58	40	37	36	This study

**Figure 8:**
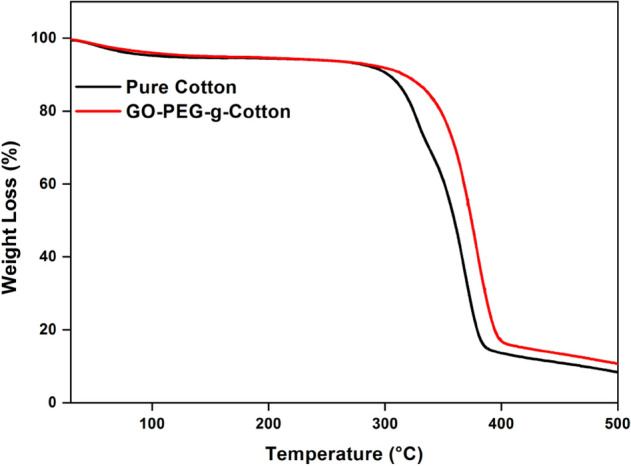
TGA analysis of pure cotton and GO-PEG-g-Cotton.

Figure 8 illustrates the decomposition behavior of prepared cotton samples using TGA. Below 100 °C, both cotton samples experienced an initial weight loss of 8% which may be due to moisture removal from pure cotton. The pure cotton sample exhibited stability up to 300 °C. The increase in temperature above 300 °C resulted in significant weight loss due to the α-cellulose decomposition of cotton. PEG-6000 is stable up to 350 °C, and subsequent increases in temperature cause it to continuously decompose^56,57^. In GO-PEG-g-Cotton, the PEG chemical grafting on the cotton backbone contributed to increased carbon–carbon and ester bonds, resulting in its better heat stability up to 320 °C.

### Thermal conductivity measurement of prepared cotton samples

Cotton and PEG side chains are both organic materials with poor thermal conductivity, hence they have mediocre heat absorption capacities in hot environments. The thermal conductivity of cotton lies between 0.026 and 0.065 W/m K^58^ while that of PEG is reported as 0.2985 W/m K^59^. As GO exhibits a high thermal conductivity of nearly 72 W/m K, GO nanosheets were deposited on PEG-g-Cotton^60^. The fabric's thermal conductivity increased with the GO deposition, allowing it to absorb more heat from the environment. The thermal conductivities for pure cotton and PEG-g-Cot were measured to be 0.045 W/m K and 0.09 W/m K, respectively, while GO deposition boosted PEG-g-Cotton thermal conductivity to 0.52 W/m K. The modified fabric's thermal conductivity after 20 cycles of washing was measured as 0.5 W/m K. This illustrates that laundering had little impact on cotton's thermal conductivity, and GO nanosheets remained trapped in the fabric polymer matrix.

### Assessment of UV protection factor of GO-PEG-g-Cotton

**Figure 9:**
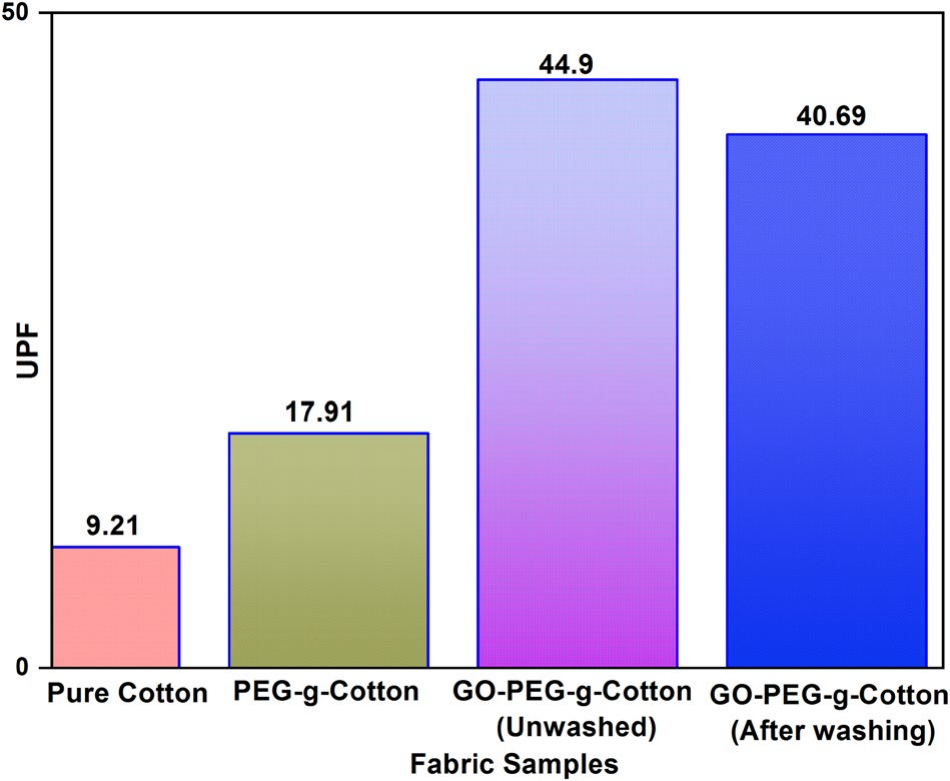
Assessment of ultra-violet protection factor of fabric samples.

The need for garments with UV protection has surged due to the ozone layer's depletion. Skin tanning, wrinkles, photoaging, and skin cancer are just a few of the major health risks that UV radiation can produce. The GO loading on the textile substrate can enhance the fabric's UV protection functionality along with an improvement in its thermal conductivity. The research is well-documented regarding GO's deposition on textiles for blocking harmful UV rays^27,61–63^. UPF for both pure cotton, PEG-g-Cotton, and GO-PEG-g-Cotton were measured, as shown in Fig. 9. UPF for PEG-g-Cotton and the pure cotton fabric were found as 9.21 and 17.91, respectively. Since, GO deposition made the fabric UV active, GO-PEG-g-Cotton exhibited the UPF of 44.9 and 40.69, before and after laundering, respectively. Thus the GO deposition enhanced the UV protection functionality of PEG-g-Cotton. Further, the UPF values before and after washing show no discernible difference, indicating that the finished cloth has good washing endurance.

### Effect on mechanical properties of GO-PEG-g-Cotton

Table 2 illustrates the effect of grafting on fabric mechanical properties. Pure and GO-PEG-g-Cotton were found to have GSM of 132 and 162, respectively. PEG grafting and GO depositions were attributed to an increase in the GSM of GO-PEG-g-Cotton. While pure cotton showed a tensile strength of 102 N, the GO-PEG-g-Cotton fabric's tensile strength was measured as 122 N. Additionally, GO-PEG-g-Cotton exhibited abrasion resistance up to 39,000 rubs while pure cotton fabric only recorded a resistance of 34,000 rubs. Noteworthy augments in tensile strength and abrading resistance of the GO-PEG-g-Cotton due to chemical grafting of PEG. The grafting process resulted in the introduction of extra carbon–carbon and ester linkages in the cotton polymeric backbone, thus providing it with improved mechanical properties.

**Table 2:** Effect of modification on cotton intrinsic properties.

Properties	Standards	Samples	
		Pure cotton	GO-PEG-g-Cotton
GSM	ASTM D-3776	132	162
Tensile strength (N)	ASTM D-5034	102	122
Abrasion resistance (number of rubs)	ASTM D-4685	34,000	39,000

### Significance and future prospective

GO-PEG-g-Cotton has a high thermal energy storage capacity, improved thermal conductivity, thermal stability, and superior UV protection. The product has huge potential for application in technical textiles and daily wear products. PEG will become an integral part of the textile, blankets, and so on. It can be used as a component of fabric. The functionalized cotton in the tape form can be employed for the cooling of electronic components, circuitry, antenna collectors, and connectors. Smart damping fabrics are used in automobiles in seat belts. This can be designed with PEG as a part of the fiber. In any case, the technology for integration is to be made ready. There is the concept of dual functional PEG-based PCM, wherein, vibration damping is possible at a certain stage of PCM. That should be taken into consideration while designing vibration-protective textiles. In all the cases the overwhelming possibility exists for PEG in varied molecular weights. There are many high-tech products derived from polyurethane resin. If a part of the diol is replaced by PEG, it will add smart characteristics to the product. The research in the present manuscript is one attempt for progressing toward the future.

## Conclusions

Cotton was successfully grafted with carboxyl-terminated PEG. ATR- FTIR, Raman spectroscopy, XRD, XPS, and FE-SEM confirmed the formation of GO-PEG-g-Cotton. The DSC revealed that melting and crystallization peak in GO-PEG-g-Cotton were observed around 58 °C and 40 °C respectively, with heating and cooling enthalpy of 37 and 36 J/g. The TGA of GO-PEG-g-Cotton exhibited good thermal stability. The modified cotton showed an increase in thermal conductivity up to 0.52 W/m K, while pure and PEG-g-Cotton conductivity was measured as 0.045 W/m K and 0.09 W/m K, respectively. The UPF of pure cotton and GO-PEG-g-Cotton (before and after washing) were measured as 9.21, 44.9, and 40.69 respectively. The fabric is highly capable of blocking harmful UV rays and possesses better washing durability. A notable improvement in tensile strength and abrasion resistance of modified fabric was found. Since the grafting of PEG onto cotton is non-toxic, eco-friendly, and less costly, the introduction of S-SPCMs in textile products is a promising approach that can be highly beneficial for consumers. The product has huge potential for application in technical textiles and daily wear products.

## Supplementary Information

Supplementary Information.

## Data Availability

The datasets generated during and/or analysed during the current study are available from the corresponding author on reasonable request.
