# Supplementary material for: Cotton functionalized with polyethylene glycol and graphene oxide for dual thermoregulating and UV-protection applications
Source: Sci Rep. 2023 Apr 11;13:5923. doi: 10.1038/s41598-023-31415-z (PMC10090063; doi:10.1038/s41598-023-31415-z)
Supplement: Supplementary file 1 — Supplementary Information. [file 41598_2023_31415_MOESM1_ESM.docx]

**Supplementary Material**

**1. EDX Analysis : a) Pure Cotton b) PEG Grafted Cotton c) GO deposited-PEG grafted cotton (before washing) d) ) GO deposited-PEG grafted cotton (after washing)**

**a) Pure Cotton**


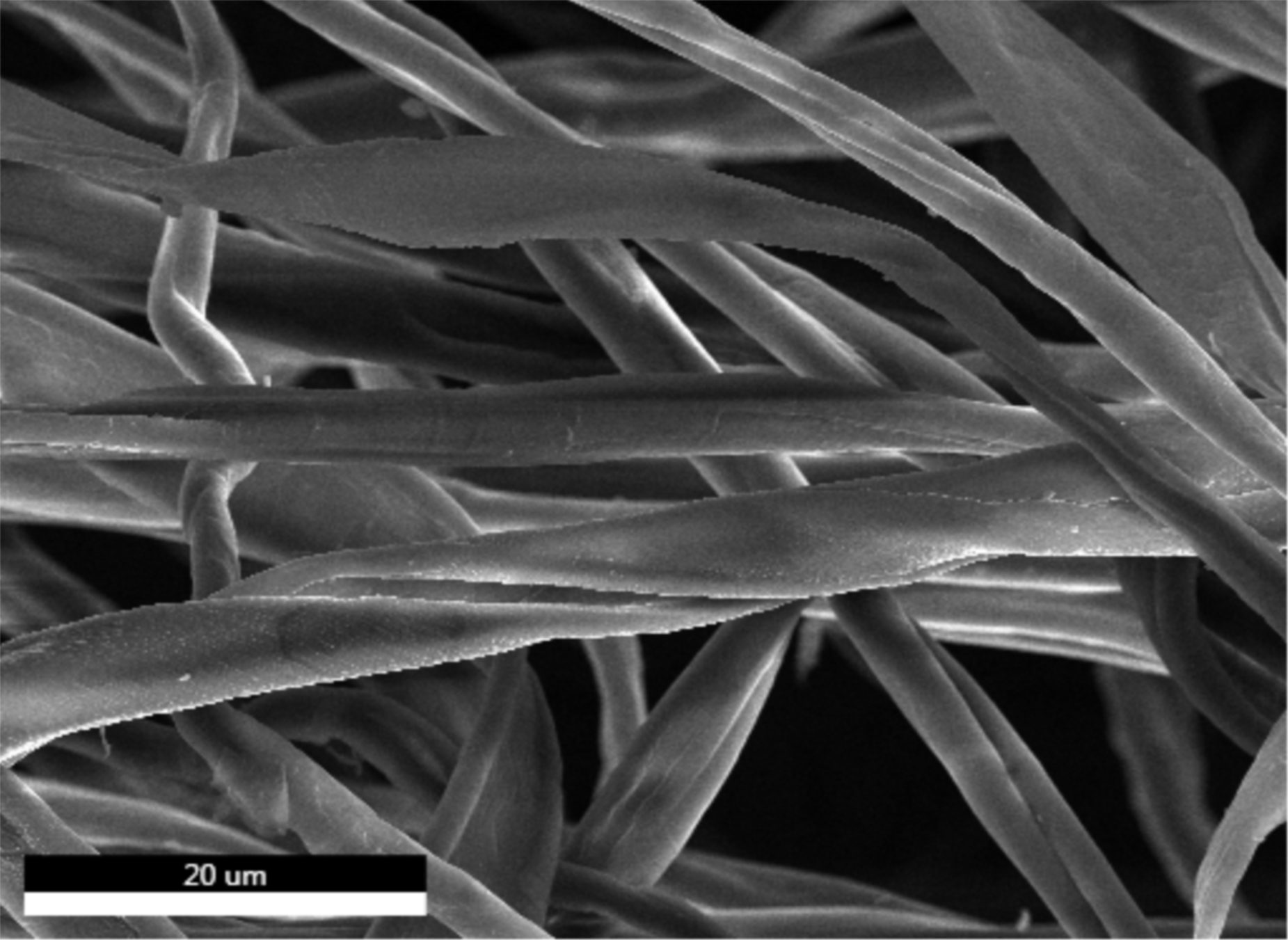


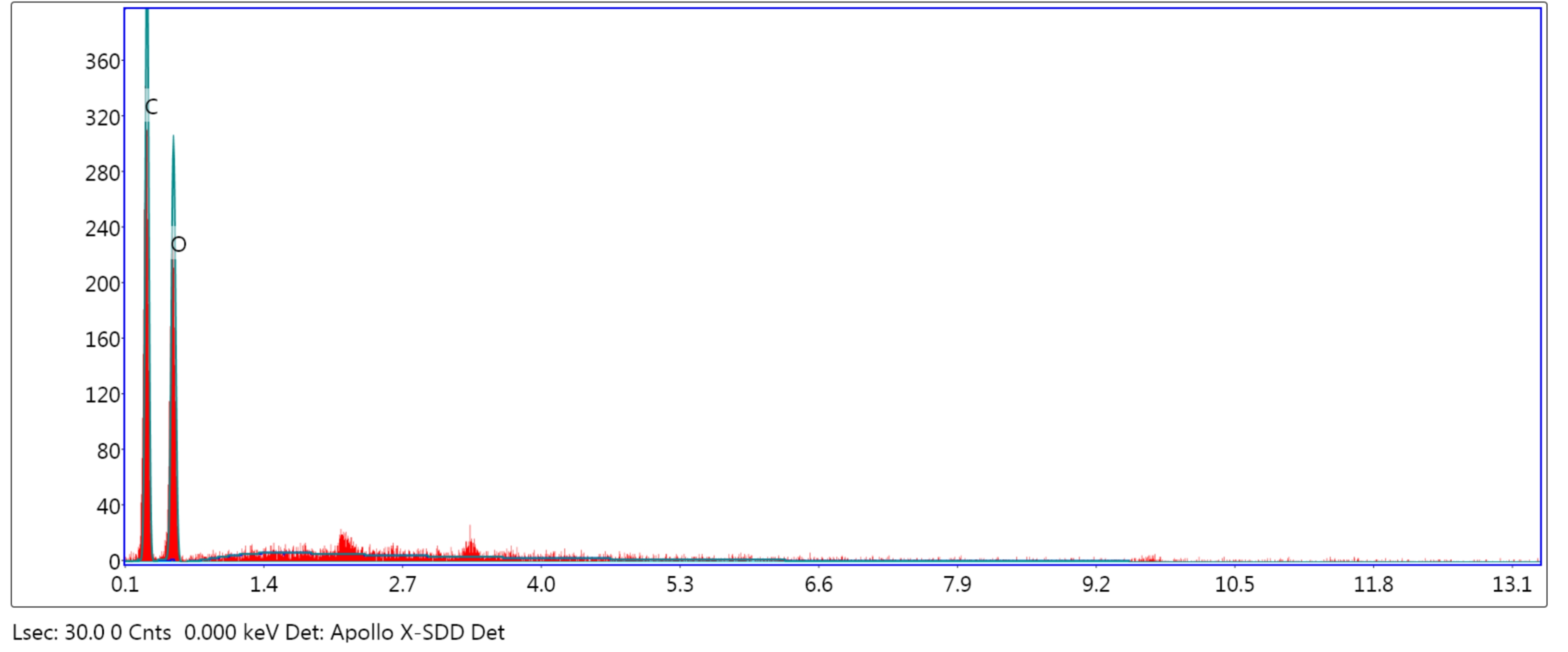


| **Element** | **Weight %** | **Atomic %** | **Net Int.** | **Error %** | **Kratio** | **Z** | **R** | **A** | **F** |
| --- | --- | --- | --- | --- | --- | --- | --- | --- | --- |
| C K | 50.20 | 57.31 | 121.32 | 6.78 | 0.30 | 1.02 | 0.99 | 0.59 | 1 |
| O K | 49.80 | 42.69 | 97.32 | 10.99 | 0.10 | 0.98 | 1.01 | 0.2 | 1 |

**b) PEG Grafted Cotton**

**
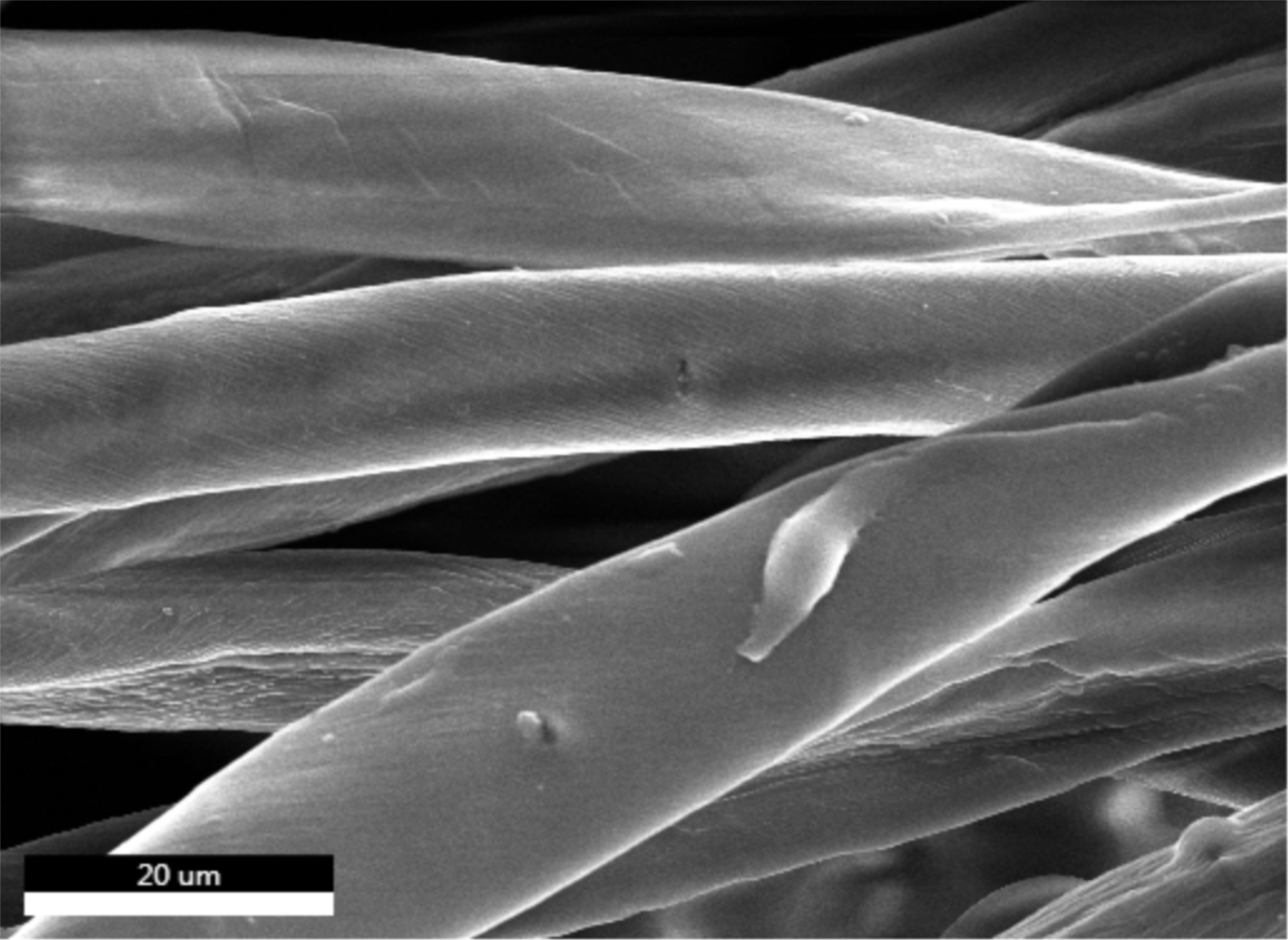
**

**
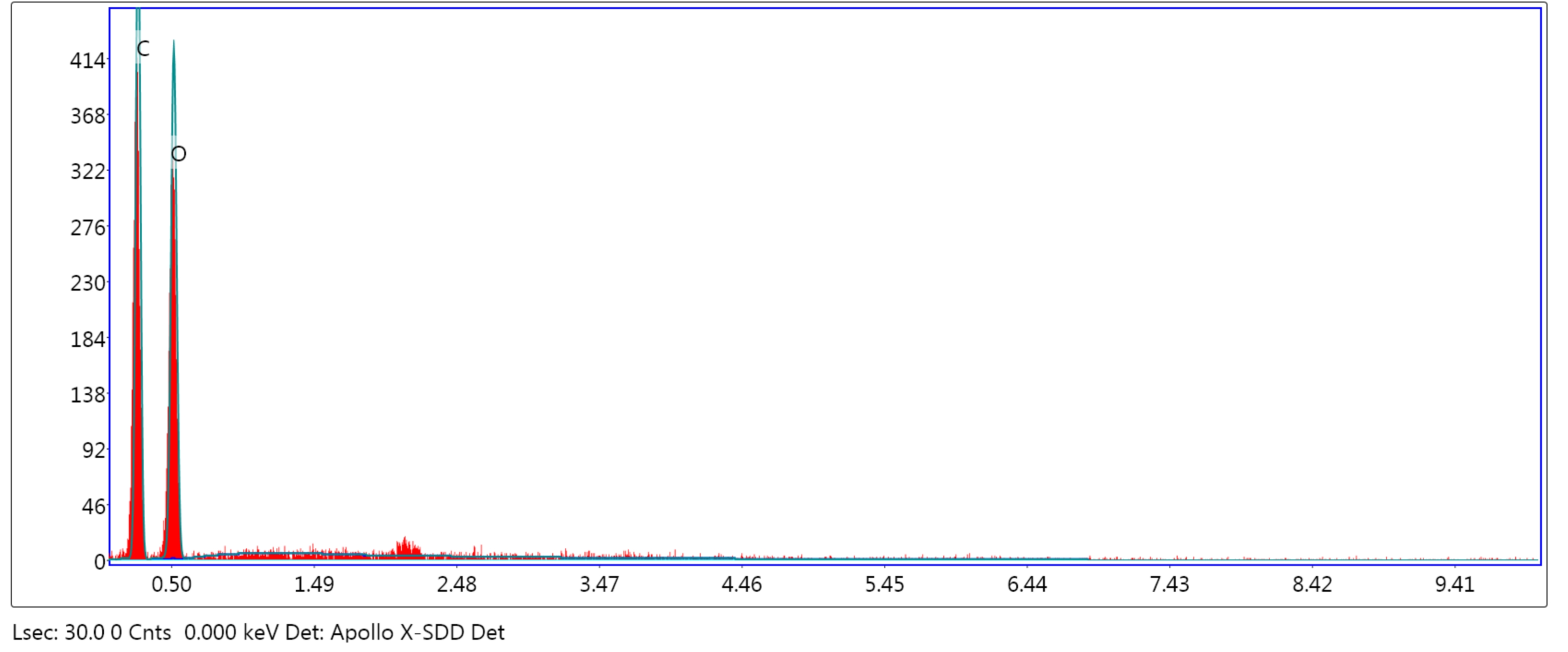
**

| **Element** | **Weight %** | **Atomic %** | **Net Int.** | **Error %** | **Kratio** | **Z** | **R** | **A** | **F** |
| --- | --- | --- | --- | --- | --- | --- | --- | --- | --- |
| C K | 48.48 | 55.62 | 147.28 | 6.32 | 0.32 | 1.03 | 0.99 | 0.64 | 1 |
| O K | 51.52 | 44.38 | 136.44 | 10.06 | 0.15 | 0.97 | 1.01 | 0.29 | 1 |

**c) GO deposited-PEG grafted cotton (before washing)**

**
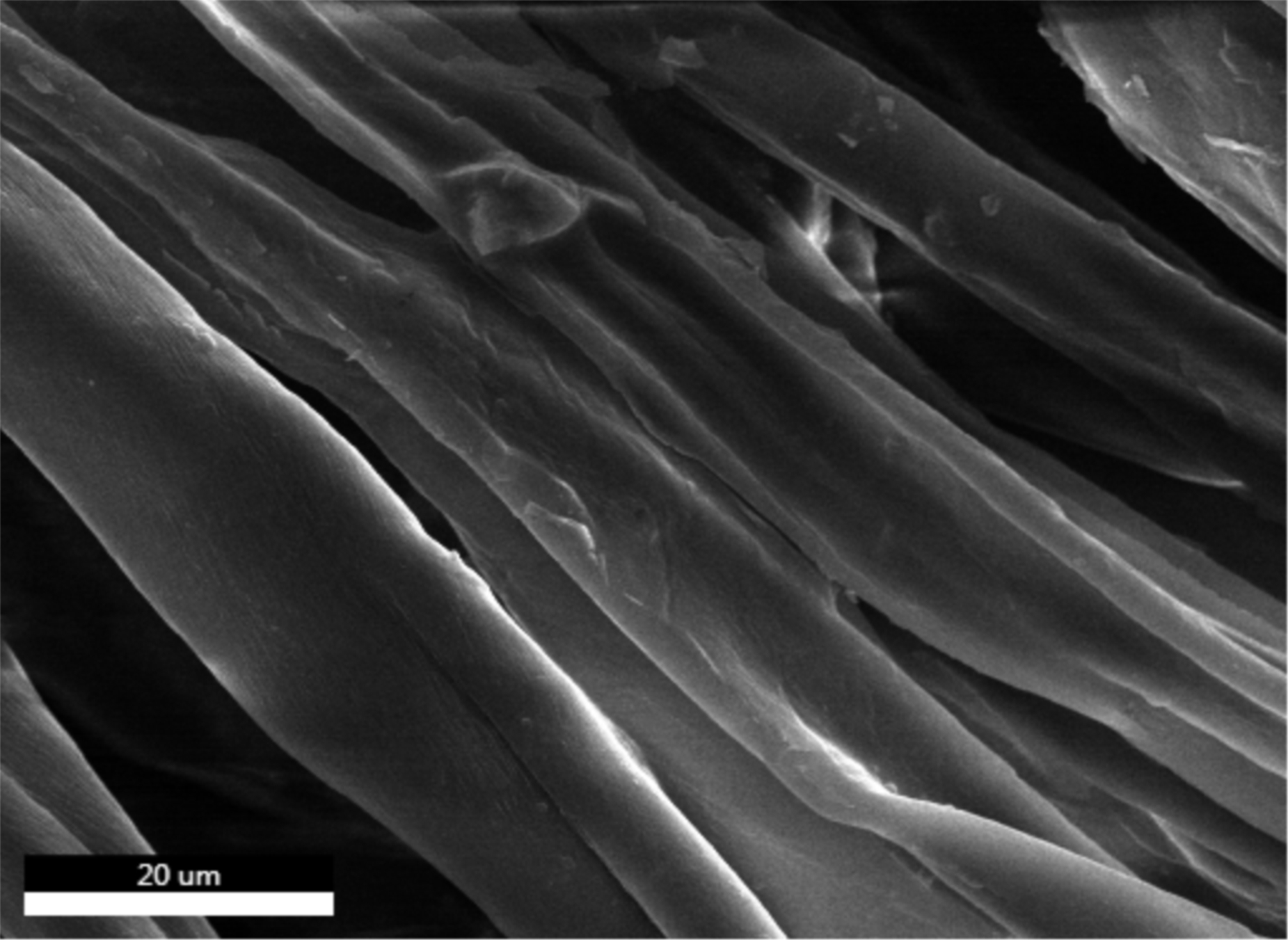
**

**
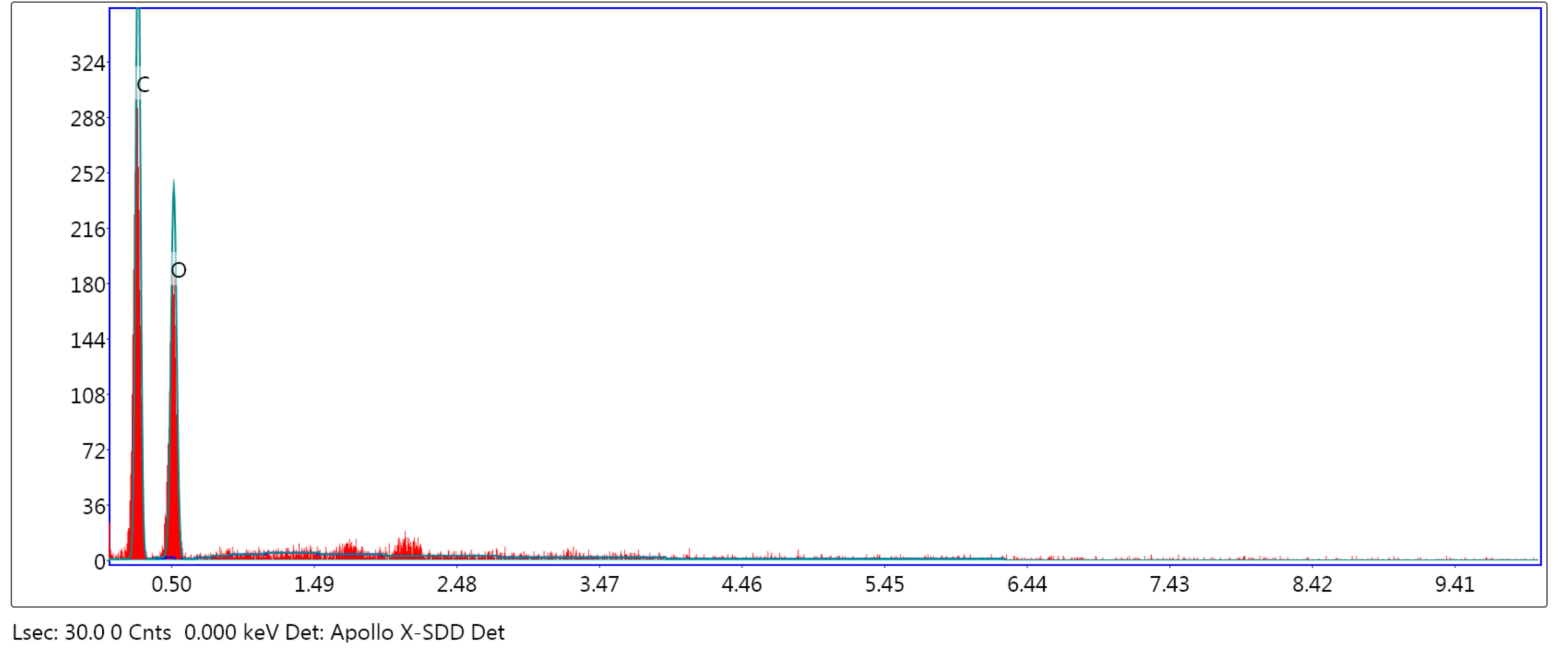
**

| **Element** | **Weight %** | **Atomic %** | **Net Int.** | **Error %** | **Kratio** | **Z** | **R** | **A** | **F** |
| --- | --- | --- | --- | --- | --- | --- | --- | --- | --- |
| C K | 53.39 | 60.41 | 116.66 | 6.29 | 0.37 | 1.02 | 0.99 | 0.67 | 1 |
| O K | 46.61 | 39.59 | 78.26 | 11.02 | 0.12 | 0.97 | 1.01 | 0.27 | 1 |

**d) GO deposited-PEG grafted cotton (after washing)**


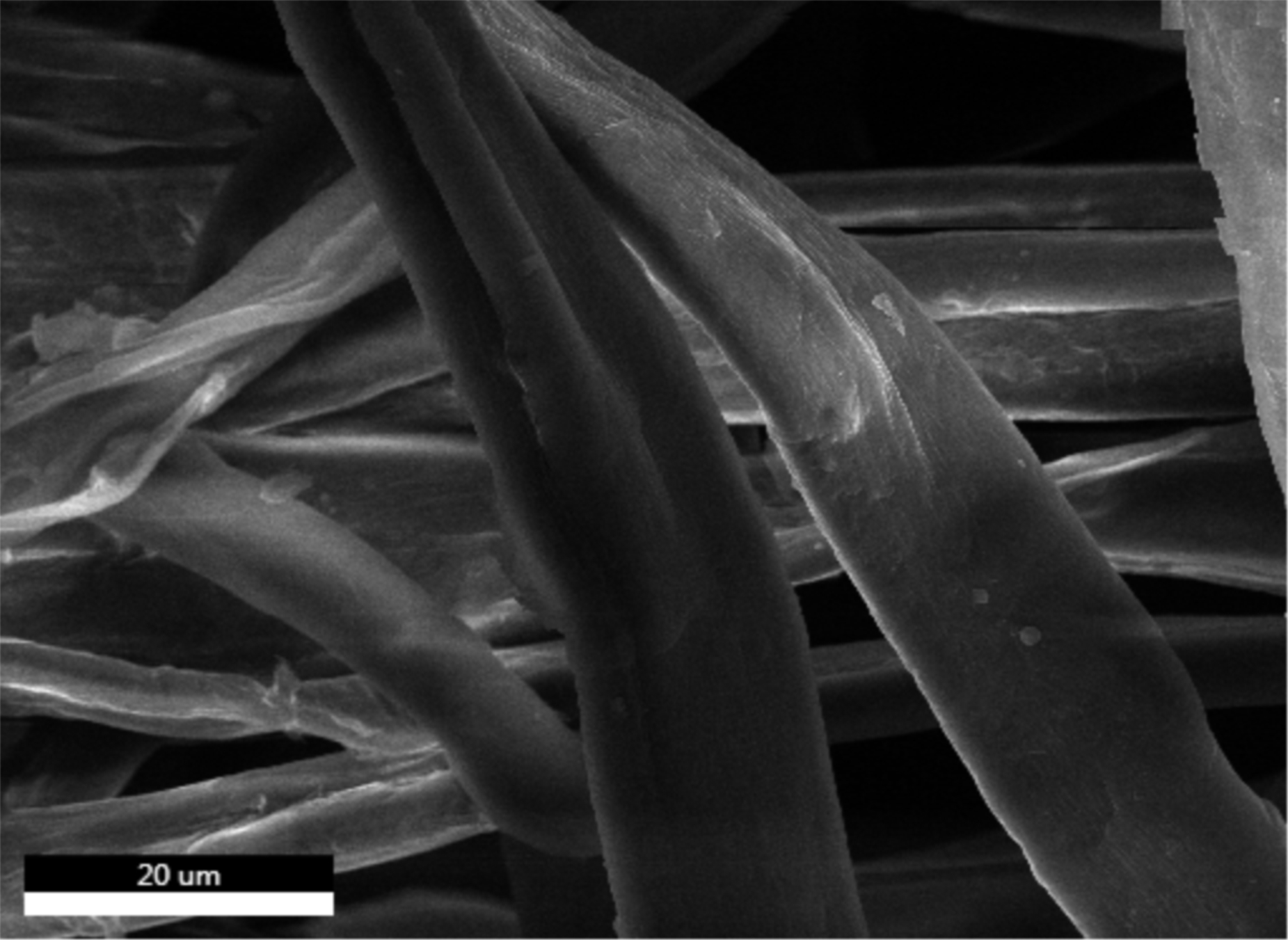


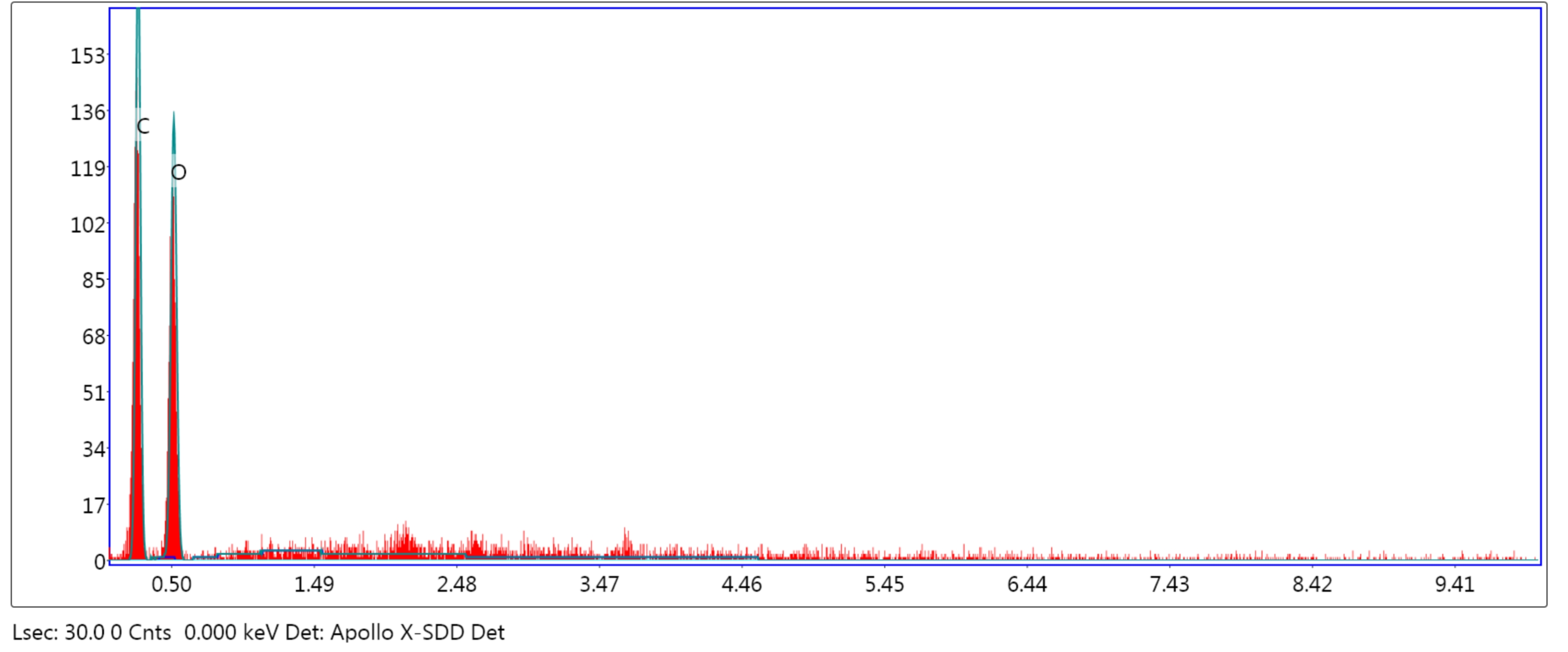


| **Element** | **Weight %** | **Atomic %** | **Net Int.** | **Error %** | **Kratio** | **Z** | **R** | **A** | **F** |
| --- | --- | --- | --- | --- | --- | --- | --- | --- | --- |
| C K | 49.91 | 57.03 | 50.91 | 7.44 | 0.33 | 1.02 | 0.99 | 0.65 | 1 |
| O K | 50.09 | 42.97 | 42.94 | 11.66 | 0.14 | 0.97 | 1.01 | 0.28 | 1 |
